# Maternal embryonic leucine zipper kinase serves as a poor prognosis marker and therapeutic target in gastric cancer

**DOI:** 10.18632/oncotarget.6673

**Published:** 2015-12-19

**Authors:** Shen Li, Ziyu Li, Ting Guo, Xiao-Fang Xing, Xiaojing Cheng, Hong Du, Xian-Zi Wen, Jia-Fu Ji

**Affiliations:** ^1^ Key Laboratory of Carcinogenesis and Translational Research (Ministry of Education), Division of Gastrointestinal Cancer Translational Research Laboratory, Peking University Cancer Hospital & Institute, Beijing, China; ^2^ Department of Gastrointestinal Surgery, Peking University Cancer Hospital & Institute, Beijing, China

**Keywords:** MELK, gastric cancer, prognosis, metastasis, PDX

## Abstract

Maternal embryonic leucine zipper kinase (MELK) is upregulated in a variety of human tumors, and is considered an attractive molecular target for cancer treatment. We characterized the expression of MELK in gastric cancer (GC) and measured the effects of reducing MELK mRNA levels and protein activity on GC growth. MELK was frequently overexpressed in primary GCs, and higher MELK levels correlated with worse clinical outcomes. Reducing MELK expression or inhibiting kinase activity resulted in growth inhibition, G2/M arrest, apoptosis and suppression of invasive capability of GC cells *in vitro* and *in vivo*. MELK knockdown led to alteration of epithelial mesenchymal transition (EMT)-associated proteins. Furthermore, targeting treatment with OTSSP167 in GC patient-derived xenograft (PDX) models had anticancer effects. Thus, MELK promotes cell growth and invasiveness by inhibiting apoptosis and promoting G2/M transition and EMT in GC. These results suggest that MELK may be a promising target for GC treatment.

## INTRODUCTION

Gastric cancer is the fourth most common malignant tumor and the second leading cause of cancer-related deaths [1]. Despite improvements in detection and management, the 5-year survival rate for GC remains low [2]. Because patients in the early stages of GC are either asymptomatic or report only nonspecific symptoms, by the time of diagnosis the tumor has often progressed to an advanced stage or has even metastasized to distant organs. Metastasis is the most common cause of death in patients with GC [3]. Further study of the molecular mechanisms of GC development and progression may help identify new molecular targets for more effective therapies.

Molecules that are uniquely overexpressed in cancer cells are ideal targets for the development of anticancer drugs, and treatments focusing on specific molecular targets often have fewer negative side effects. Protein kinases have emerged as the most important targets for drug discovery because of their critical roles in regulating cell growth and survival. Data collected previously using an Affimetrix HG-133 array showed a 3.84-fold increase in *MELK* expression in 79 GC tissues as compared to 24 non-cancerous tissues, making it one of the most upregulated genes in GC (*p* < 0.05) (unpublished data).

Maternal embryonic leucine zipper kinase (MELK), also known as murine protein K38 (MPK38) and Eg3 protein, was firstly identified in unfertilized eggs and is now known to be a member of the AMP-activated Ser/Thr protein kinase family [4–6]. During later stages of embryogenesis, *MELK* expression is restricted to areas where mesenchymal-epithelial interactions are taking place [7]. In normal adult tissues, *MELK* is expressed only in the testis and at very low levels in the thymus and small intestine [4, 8]. MELK participates in multiple cellular processes, including cell cycle progression, cell proliferation, apoptosis, RNA processing, and cell migration [8–13].

Overexpression of MELK has been detected in a variety of human tumors, including breast cancer, glioblastoma, prostate cancer, colorectal cancer, and acute myeloid leukemia [8, 14–17]. Furthermore, high levels of MELK expression correlate with poor prognoses in patients with breast cancer and glioma [18, 19]. Recent research has explored the role of MELK in cancer stem cell maintenance and survival [14, 20, 21]. However, little is known about the relevance of this kinase in gastric malignancies. In this study, we characterized the expression of MELK in GC patients and investigated its role in GC pathogenesis. We quantified MELK expression in both human GC cell lines and primary GC tissues, and examined the relationships between MELK expression and metastasis, depth of invasion, and poor prognosis in GC patients. MELK mRNA and protein activity were reduced using shRNA and OTSSP167, a small molecule inhibitor of MELK kinase activity [22], and the effects on GC cell growth, G2/M arrest, apoptosis, invasive capability of GC cells, and as well as decreased metastatic colonization in xenograft models were measured.

## RESULTS

### Expression of MELK in human GC cell lines and primary GCs

**Figure 1:**
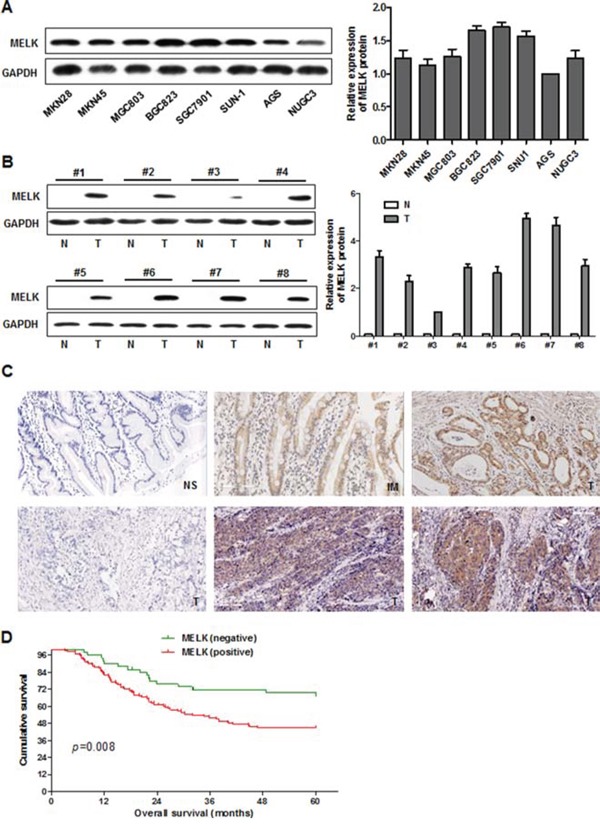
MELK expression in cultured GC cells and primary GC tissues, and survival in patients with GC **A.** Western blotting analysis of MELK in GC cell lines. **B.** MELK expression in primary GC (T) and distal surgical margin normal mucosa (N) were examined by Western blotting. **C.** Expression of MELK by immunohistochemical staining. Original magnification: 200x. NS: normal stomach; IM: intestinal metaplasia; T: gastric cancer. **D.** Kaplan-Meier survival curves of overall survival for all patients with MELK-negative vs. -positive GC tissue.

We assessed the levels of *MELK* mRNA and MELK protein by polymerase chain reaction (PCR) and Western blotting, respectively, in 8 human GC cell lines. As shown in Fig. S1 and 1A, *MELK* mRNA was highly expressed in all cell lines and protein levels correlated well with mRNA levels. MELK protein levels were also analyzed in frozen GC specimens and corresponding distal surgical margin tissue from 8 patients using Western blotting (Fig. 1B). MELK was detected in all of the tumor tissues but not in any of the distal surgical margin samples, indicating that MELK is upregulated in GC tissues compared to normal stomach mucosa.

Next we measured the expression of MELK in primary GC tissues using immunohistochemistry (IHC). MELK was seldom expressed in normal stomach mucosa, but was highly expressed in the cytoplasm of GC tissues (Fig. 1C). MELK was expressed in 71.9% (128/178) of the GC tumor tissue samples. Of the 104 cases that had adjacent noncancerous mucosa present in the same section, MELK was expressed more frequently in the cancer lesion than the corresponding noncancerous mucosa (68.3% vs. 28.8%, *p*=0.036) (Table S1). In addition, MELK was expressed in 66.7% (12/18) of gastric intestinal metaplasia (IM) samples, a precancerous lesion of the stomach present in some GC specimens (Fig. 1C).

### Overexpression of MELK was associated with lymph node involvement, distant metastasis, and poor prognosis in patients with GC

To clarify the role of MELK in gastric carcinogenesis, we analyzed the association between MELK expression and clinicopathological characteristics in GC patients. As shown in Table 1, GC patients with lymph node involvement and distant metastasis exhibited higher MELK expression rates than patients without these characteristics (77.1% vs. 57.4%, *p*=0.010; 91.7% vs. 68.8%, *p*=0.024, respectively). Likewise, a larger portion of advanced GC patient tissues expressed MELK compared to early gastric carcinoma tissues (T1 vs. T2-4, *p*=0.004), and expression tended to increase in more advanced TNM stages *(p=*0.056).

**Table 1:** Relationship between MELK expression and clinicopathological features in patients with gastric cancer

	MELK expression		χ^2^	*P*^a^
	Negative n (%)	Positive n (%)		
Gender				
Male	35 (26.3)	98 (73.7)	0.820	0.365
Female	15 (33.3)	30 (66.7)		
Age, years				
≤ 60	35 (25.4)	103 (74.6)	2.262	0.075
> 60	15 (37.5)	25 (62.5)		
Tumor location				
Cardiac	8 (29.6)	19 (70.4)	0.037	0.874
Non cardiac	42 (27.8)	109 (72.2)		
Tumor size (cm)				
≤ 4	28 (28.6)	70 (71.4)	0.025	0.874
> 4	22 (27.5)	58 (72.5)		
Lauren				
Intestinal/mixed	22 (20.4)	86 (79.6)	8.102	0.004
Diffuse	28 (40.0)	42 (60.0)		
Vascular invasion				
Absent	27 (29.3)	65 (70.7)	0.149	0.699
Present	23 (26.7)	63 (73.3)		
Depth of invasion				
T1	9 (60.0)	6 (40.0)	8.257	0.004
T2-4	41 (22.9)	122 (77.1)		
Lymph node				
No	20 (42.6)	27 (57.4)	6.614	0.010
Yes	30 (22.9)	101 (77.1)		
Distant metastasis				
M0	48 (31.2)	106 (68.8)	5.360	0.024
M1	2 (8.30)	22 (91.7)		
TNM stage				
I	10 (52.6)	9 (47.4)	4.304	0.056^b^
II	14 (27.5)	37 (72.5)		
III	21 (25.0)	63 (75.0)		
IV	5 (20.8)	19 (79.2)		

a Chi-square test,

b Kruskal-Wallis Test;

- negative; + positive

Multivariate analysis of 5-year overall survival (OS) showed that MELK expression was an independent marker for poor prognosis (*p*=0.017) (Table 2). Kaplan-Meier survival curves showed that OS was worse in GC patients with MELK expression compared to patients showing no expression (*p* = 0.008, Fig. 1D).

**Table 2:** Results of univariate and multivariate Cox proportional-hazards regression analysis for overall survival of GC patients

Parameter	Univariate	*P*	Multivariate	*P*
	5-year survival rate (%) (mean± S.E)		RR 95% CI	
Tumor size (cm)		0.008	1.380 (0.892-2.136)	0.148
≤ 4	60.0±0.050			
> 4	40.2±0.056			
Vascular invasion		0.001	1.198 (0.748-1.919)	0.453
Absent	62.0±0.051			
Present	39.3±0.054			
Depth of invasion		0.001	1.711 (1.125-2.602)	0.012
T1	100±0.000			
T2-4	46.6±0.039			
Lymph node involvement		0.000	1.503 (1.201-1.811)	0.000
No	74.5±0.064			
Yes	42.6±0.044			
Distant metastasis		0.000	4.588 (2.572-8.182)	0.000
M0	57.3±0.040			
M1	0.00±0.000			
MELK expression		0.008	1.950 (1.129-3.368)	0.017
Negative	67.5±0.067			
Positive	44.8±0.044			
Gender		0.154	-	-
Male	47.8 ±0.044			
Female	61.3 ±0.074			
Age, years		0.679	-	-
≤ 60	47.4±0.081			
> 60	47.4±0.081			

RR, relative risk; CI, confidence interval.

### MELK Knockdown attenuated GC cell migration and invasion both *in vitro* and *in vivo*

**Figure 2:**
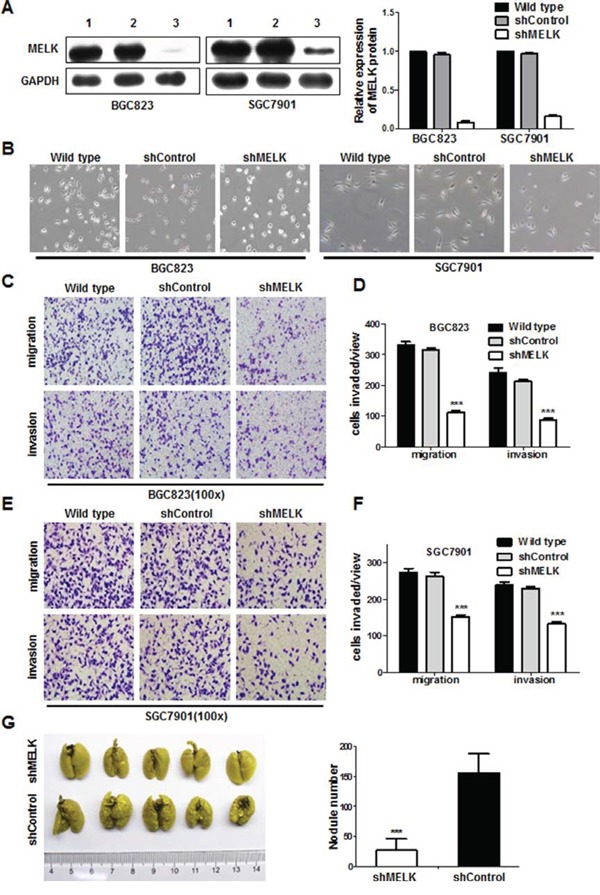
MELK knockdown attenuated migration and invasion of GC cells *in vitro* and *in vivo* **A.** MELK protein expression. Lane 1, wild type; Lane 2, scrambled shControl; Lane 3, shMELK. **B.** Morphologic changes in GC cells in response to MELK knockdown. Original magnification: ×100. **C-F.** BGC823 and SGC7901 cells were assayed for their invasive capability on Matrigel or Boyden chambers. Bars represent the mean ± SD of three independent experiments. **G.** The effect of MELK on metastatic colonization through blood circulation. *p< 0.05, **p< 0.01, _***_p< 0.001.

Since MELK was overexpressed in all GC cell lines examined, we used a loss of function approach in two GC cell types (BGC823 and SGC7901) to determine the role of MELK in gastric tumorigenesis. Knockdown of *MELK* mRNA expression following infection with a lentivirus expressing anti-*MELK* small hairpin RNA (shRNA) was confirmed by Western blotting (Fig. 2A); shRNA treatment also reduced, but did not eliminate, MELK protein expression. *MELK* knockdown resulted in morphological changes in both BGC823 and SGC7901 cells (Fig. 2B). Since *MELK* expression was associated with lymph node and distant metastasis in patients with primary GC, it is possible that MELK endows GC cells with invasive behavior. Indeed, wound healing, transwell, and Matrigel invasion assays showed that both BGC823-shMELK and SGC7901-shMELK cells displayed decreased migration and invasion as compared to their respective shControl cells (Fig. 2C-2F, Fig. S2, *p*<0.05).

Next, to demonstrate the effect of MELK on metastatic colonization, BGC823-shMELK cells and BGC823-shControl cells were injected into athymic nude mice via the tail vein. Metastatic potential was assessed by counting colonized tumor nodules in the lungs of these mice. BGC823-shMELK-injected mice had fewer lung tumor nodules compared to BGC823-shControl-injected mice (Fig. 2G). Collectively, these data indicate that MELK strongly promotes cell migration and invasion in GC.

### Targeting MELK reduced GC cell proliferation both *in vitro* and *in vivo*

**Figure 3:**
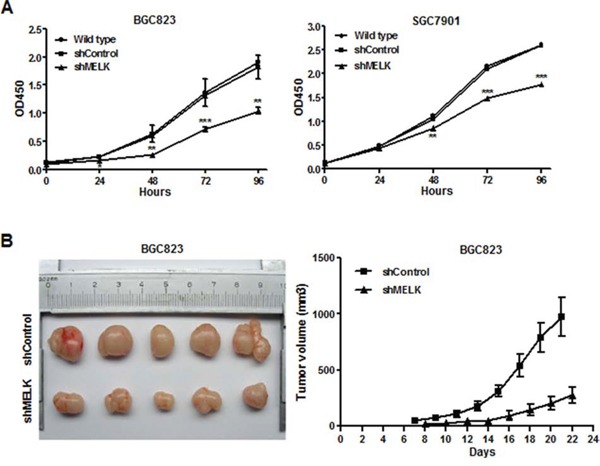
Effects of MELK knockdown on GC cell growth *in vitro* and *in vivo* **A.** Cell proliferation assay using a Cell Counting Kit-8. **B.** Photograph showing tumor formation in nude mice injected with BGC823-shContral and shMELK, as well as tumor growth curve. The data are shown as mean ± SD. *p< 0.05, **p <0.01, ***p< 0.001.

The effects of MELK knockdown on the proliferation of BGC823 and SGC7901 cells were examined using Cell Counting Kit-8. As shown in Fig. 3A, shRNA-induced *MELK* knockdown inhibited cell growth. OTSSP167, a small molecular inhibitor (SMI) of MELK, also markedly suppressed proliferation of BGC823 cells in a dose-dependent manner (Fig. S4). The IC50 of OTSSP167 in these cells was 25 nM as determined using an xCELLigence real-time cell analyzer (RTCA).

In a xenograft experiment, BGC823-shControl and BGC823-shMELK cells were subcutaneously injected into the right hind legs of the nude mice. Tumors were smaller and their growth was slower in the BGC823-shMELK-injected group compared to the BGC823-shControl-injected group (Fig. 3B). Thus, *MELK* knockdown inhibited GC cell viability and proliferation both *in vitro* and *in vivo*.

### Inhibition of MELK induced apoptosis and G2/M arrest in GC cells

**Figure 4:**
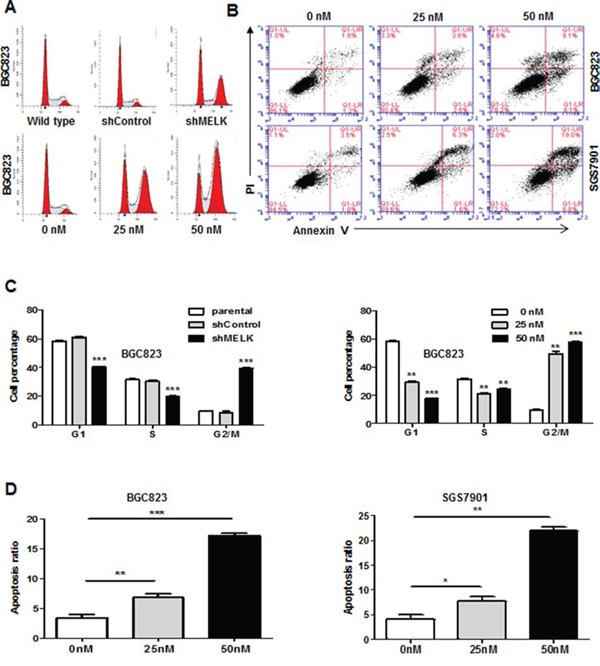
Targeting *MELK* expression or MELK kinase activity results in G2/M arrest and apoptosis **A.** Cell cycle analysis by Flow cytometry. **B.** Apoptosis assessed by Annexin V and PI staining in BGC823 and SGC7901 cells treated with different concentrations of OTSSP167. **C.** Histogram of cell cycle distribution. Bars, mean ± SD of three independent experiments. **D.** Histogram of cell apoptosis. Bars, mean ± SD of three independent experiments. *p < 0.05, **p < 0.01, ***p < 0.001.

To determine whether MELK inhibits cell growth by triggering apoptosis or suppressing DNA synthesis, we monitored cell cycle progression after *MELK* knockdown using Fluorescence-activated cell sorting (FACS). A higher proportion of cells infected with shMELK lentivirus were arrested in the G2/M phase of the cell cycle compared to those infected with scrambled shControl (39.67% vs. 8.66% in BGC823 cells, and 17.57% vs. 11.60% in SGC7901 cells, respectively, Fig. 4A upper panel, Fig. 4C left panel, and Fig. S3A). The increase in the G2/M phase population was accompanied by a decrease in the number of cells in the S-phase. Targeting MELK enzymatic activity with 25 nM and 50 nM OTSSP167 also increased the G2/M phase population compared to vehicle treatment (57.88% vs. 9.83% in BGC823 cells, and 21.37% vs. 9.83% in SGC7901 cells, respectively, Fig. 4A lower panel, Fig. 4C right panel, and Fig. S3B). Apoptosis was assessed by annexin V and PI staining in BGC823 and SGC7901 cells treated with vehicle or 25 nM or 50 nM OTSSP167. OTSSP167 treatment increased apoptosis in a dose-depended manner compared to vehicle treatment (16.9% vs. 4.2% for BGC823 and 23.47% vs. 4.7% for SGC7901, *p*<0.05) (Fig. 4B and 4D). These data suggest that MELK promotes GC cell survival and proliferation by accelerating transition out of the G2/M phase and by reducing apoptosis.

### MELK may facilitate epithelial–mesenchymal transition (EMT) in GC cells

**Figure 5:**
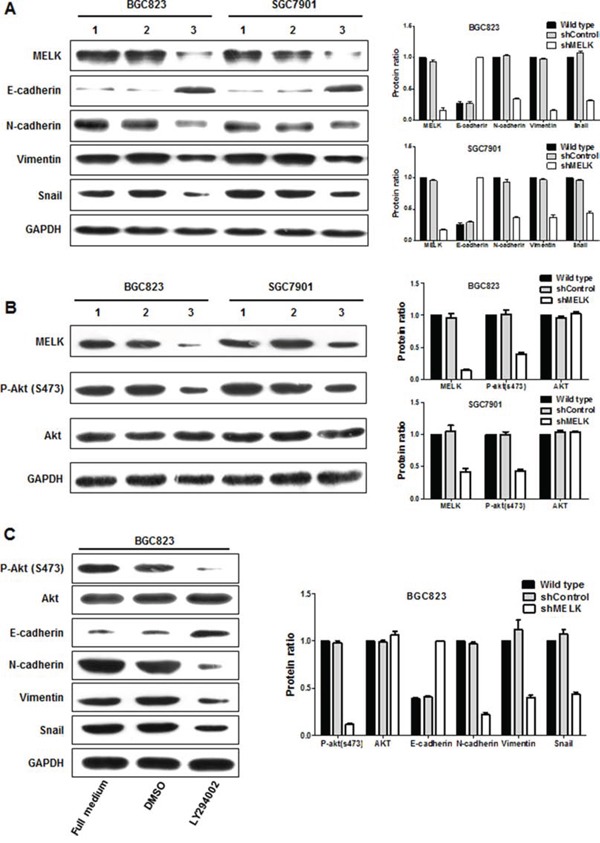
MELK regulates the expression of EMT-associated proteins and AKT activity **A and B.** Expression profiles of EMT components in response to MELK knockdown determined using Western blotting. Lane 1, wild type; Lane 2, scrambled shControl; Lane 3, shMELK. © Altered expression of EMT-associated proteins and Akt activity in BGC823 cells treated with LY294002.

Enhanced cell migration and invasion capabilities are important consequences of EMT, an early event in tumor metastasis. Here, we found that MELK depletion in BGC823 and SGC7901 cells changed cell morphology from an elongated spindle-like appearance to a cobblestone-like appearance (Fig. 2B). Morphological alteration is one of the main features of EMT. Therefore, we examined the expression of markers associated with EMT by Western blotting. MELK depletion upregulated E-cadherin and downregulated N-cadherin, Vimentin, and Snail in both BGC823 and SGC7901 cells (Fig. 5A). Thus, MELK expression may contribute to metastasis and poor prognosis in patients with primary GCs by promoting EMT in GC cells.

### MELK activated Akt signaling via phosphorylation

Activity of the oncogenic kinase Akt is most commonly associated with GC [23], and the PI3K/Akt axis is emerging as a central promoter of EMT [24]. Both MELK and Akt are serine/threonin kinases, and it is possible that MELK might activate Akt. There were no differences in total Akt protein levels among MELK-shRNA, scrambled shControl, or wild-type cells. However, MELK knockdown decreased levels of phosphorylated Akt (Ser-473) protein compared to scrambled control-treated or wild-type cells (Fig. 5B). Treatment with LY294002, a PI3K inhibitor, similarly reduced Akt phosphorylation and altered EMT-associated protein levels in BGC823 cells (Fig. 5C). Together, these data suggest that MELK is involved in the PI3K/Akt signal cascade and that MELK may induce EMT by activating Akt. Further study is required to determine whether Akt is a direct substrate of MELK.

### Efficacy of OTSSP16 treatment in preclinical GC patient-derived xenograft (PDX) mouse models

**Figure 6:**
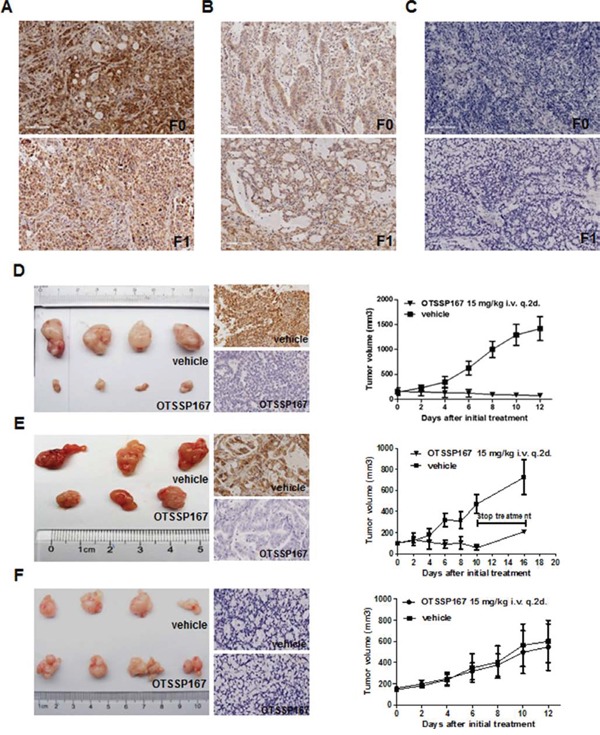
OTSSP167 reduces growth of MELK-positive GC-PDX TumorGraft **A, B and C.** IHC for MELK in patients' tumors. **A.** MELK-positive staining in the case #1 GC patient-derived TumorGraft. **B.** MELK-positive staining in the case #3 GC patient-derived TumorGraft. **C.** MELK-negative staining in the case #2 GC patient-derived TumorGraft. F0, primary GC tissue; F1, 1^st^ generation of TumorGraft. Original magnification: 200x. **D, E and F.** NOD/SCID mice bearing TumorGraft of the F3 generation were intravenously treated with OTSSP167 or vehicle control once every two days for two weeks. Tumor volume was measured every two days (right panel), and MELK expression in TumorGraft tissue after treatment was detected by IHC (middle panel). Tumor volume is shown as mean ± SD.

Two MELK-positive, and one MELK-negative, GC-PDX models were chosen from our established banks to evaluate whether MELK is an effective therapeutic target for GC *in vivo* (Fig. 6A, 6B, and 6C). Third generation PDX mice were used in this experiment. When the TumorGraft volume reached 100-200 mm^3^, the PDX mice were intravenously treated with OTSSP167 (15 mg/kg) or vehicle once every other day for two weeks. The reaction to OTSSP167 was quantified by tumor growth inhibition (TGI). In the two MELK-positive models, TGI values were 106% and 112% at the end of drug administration (Fig.6D and 6E, right panel). In the MELK-negative model, the TGI value was only 19% (Fig. 6F, right panel). MELK expression levels in the TumorGraft tissues were subsequently evaluated by IHC. In both MELK-positive cases, MELK expression was eliminated in the TumorGraft after OTSSP167 treatment but not after vehicle treatment (Fig. 6D and 6E, middle panel). These data strongly suggest that MELK might be an effective molecular target for the treatment of gastric cancer.

## DISCUSSION

Elevated MELK expression has been identified in a variety of tumors and is associated with poor prognosis in cancer patients. Our previous Affimetrix HG-133 array data showed that *MELK* is strongly upregulated in GC tumors as compared to normal gastric tissue. Here, we found that MELK was frequently overexpressed in primary GC tissues, but was not detected in normal gastric tissues. These findings are similar to the results recently reported by Du et al. [12].

We also analyzed the association between MELK expression and clinicopathologic factors. MELK expression was positively correlated with lymph node involvement, distant metastasis, and more advanced cancer stages. Furthermore, elevated MELK expression was negatively correlated with 5-year survival rate, which is consistent with the prognosis for breast cancer, prostate cancer, and glioblastoma with elevated MELK expression [8, 17, 25].

Because MELK was overexpressed in all of the GC cell lines examined, we applied two loss-of-functioning approaches. Reducing *MELK* expression using shRNA or inhibiting MELK kinase activity using OTSSP167 reduced the growth and invasiveness in GC cells. MELK expression is normally restricted to areas where mesenchymal-epithelial interactions are taking place during the later stages of embryogenesis [7], and MELK knockdown triggered morphological changes in GC cells. We therefore hypothesized that MELK may promote EMT, a key step in tumor cell migration. As expected, MELK knockdown altered expression of EMT-associated proteins. Thus, MELK expression may contribute to metastasis and poor prognosis in primary GC by promoting EMT.

TumorGrafts derived from surgical specimens conserve inter-individual diversity, microscopic fidelity, and genetic heterogeneity of the original primary tumors [26–29]. Personalized TumorGrafts have been used as an investigational platform for therapeutic decision-making that can guide treatment for individual patients, and treatments identified in this way generally elicit favorable clinical responses [30–33]. We used previously established GC-PDX models to verify the preclinical efficacy of MELK-targeting therapy in GC. We found that OTSSP167 had a strong antitumor effect in MELK-positive, but not in MELK-negative, GC-PDX models. Characterization of the immune system in *MELK* knockout mice indicates that MELK is not essential for proper bone marrow function [34]. Bone marrow toxicity is a major adverse effect of many anti-cancer therapies, especially with drugs that target mitotic kinases. MELK may therefore prove to be a promising, highly-selective target for treatment with few negative side effects.

Although MELK-targeting therapy in PDX models markedly suppressed tumor growth, it also resulted in modest body weight loss (less than 20%). However, in one of the MELK-positive tumor models, all PDX mice showed obvious body weight loss, regardless treatment. These observations might be explained by cachexia due to the severity of the primary tumors, but future studies should address the impact of treatments that reduce MELK activity on other indicators of health, including body weight.

In summary, MELK is frequently upregulated in both human GC cell lines and primary GC tissues, and its expression correlates with metastasis and a poor prognosis in GC patients. Importantly, reducing MELK expression or inhibiting kinase activity increased G2/M arrest and apoptosis, thereby inhibiting GC cell growth both *in vitro* and *in vivo*. *MELK* knockdown also inhibited invasive behavior in GC cells and decreased metastatic colonization in xenograft models. Notably, we demonstrated for the first time that MELK activity upregulates EMT. Furthermore, we showed that reducing MELK activity with OTSSP167 has anticancer effects in preclinical GC-PDX murine models. Our findings suggest that clinical evaluation of MELK-targeting therapies is warranted as a novel treatment strategy for GC patients.

## MATERIALS AND METHODS

### Cell culture and reagents

AGS, SNU1, MKN45, and 293T cell lines were purchased from ATCC (Manassas, VA, USA). MKN28 and NUGC3 cell lines were obtained from the Health Science Research Resources Bank (Tokyo, Japan). BGC823, MGC803, and SGC7901 cell lines were obtained from the Cell Research Institute (Shanghai, China). The cells were cultured in RPMI-1640 medium (GIBCO, Carlsbad, NY, USA), supplemented with 10% (v/v) fetal calf serum (GIBCO, NY, USA) and antibiotics at 37°C in a humidified 5% CO_2_ atmosphere.

Antibodies for Vimentin, E-cadherin, N-cadherin, Akt, phospho-Akt (Ser-473), ERK1/2, phospho-ERK1/2 (Thr-202/Tyr-204), Snail, and GAPDH were purchased from Cell Signaling Technology (Beverly, MA). Anti-MELK antibody and puromycin were purchased from Sigma-Aldrich (St. Louis, MO, USA). OTSSP167 was purchased from Medchem Express (Beijing, China).

### RNA isolation and polymerase chain reaction (PCR)

Total RNA was extracted using TRIzol reagent (Invitrogen, Carlsbad, CA, USA) and reverse transcribed into cDNA using M-MLV reverse transcriptase (Promega, Madison, WI, USA) according to the manufacturer's instructions. GAPDH was used as an internal control. The primer sequences used are listed below: MELK: forward primer: 5′-GCTGCAAGGTATAATTGATGGA-3′, reverse primer: 5′-CAGTAACATAATGACAGATGGGC-3′.

### Patients and gastric tissue specimens

A total of 178 paraffin-embedded GC tissues were collected from GC patients who underwent radical gastrectomy at Peking University Beijing Cancer Hospital between January 2003 and December 2007. Additionally, eight surgically removed frozen (stored at −70°C) GC samples and corresponding surgical margins collected in November 2014 were obtained from the BioBank of Beijing Cancer Hospital. All patients signed informed consent forms, and the Ethics Committee of Beijing Cancer Hospital approved tissue collection. Clinicopathological and follow-up information was obtained from patient data. Gastric cancer stage was classified according to the 2010 tumor-node metastasis (TNM) classification recommended by the American Joint Committee on Cancer (AJCC 7^th^ edition). T and N classification were assessed based on the final pathological result and M classification was determined by surgical findings. Early gastric cancer (EGC) was defined as a tumor that was confined to the mucosa or submucosa regardless of lymph node (LN) involvement. Advanced gastric cancer (AGC) was defined as a tumor that invaded the muscle proper or beyond. Overall survival (OS) was calculated beginning from the date of the initial surgery and ending either at the time of death caused by the tumor or at the date of the last follow-up. All patients were tracked until 2012. None of the patients received chemotherapy or radiation therapy prior to surgery.

This study was performed with the approval of the Ethics Committee of Peking University Beijing Cancer Hospital.

### Immunohistochemistry

Four-micrometer sections from formalin-fixed paraffin-embedded (FFPE) tissues were deparaffinized in xylene and rehydrated through graded alcohol washes. Antigen retrieval was performed by autoclaving in 0.01M citrate buffer (pH 6.0) for 3 minutes, followed by immersion in 3% hydrogen peroxide in methanol for 10 minutes to block endogenous peroxidase activity. The sections were then blocked with normal sheep serum (DAKO, Hamburg, Germany) for 90 minutes at room temperature and then incubated with MELK polyclonal antibody (Sigma-Aldrich, St. Louis, MO, USA) diluted at 1:300 overnight at 4°C. Diaminobenzidine was used as a chromogen, followed by counterstaining with hematoxylin. Samples were considered MELK-positive when 10% or more of the cancer cells had cytoplasmic MELK staining. The expression of MELK was assessed independently by two experienced pathologists who were blind to the patients' clinical outcomes. There was a high level of consistency between the two pathologists, and in the few discrepant cases (<5%) a consensus was reached after joint review.

### Lentiviral vector transduction of GC cells to stably silence MELK

The MELK expression knockdown procedure was conducted as previously described [35]. Lentivirus was produced by the co-transfection of 293T cells with a pLenti vector (pGLV3-shControl or pGLV3-shMELK) and lentiviral packaging mix (Invitrogen, Carlsbad, CA, USA) according to the manufacturer's instructions. Lentivirus-containing supernatant was harvested at 48 hours post-transfection, centrifuged, and stored at −80°C. For viral transductions, 1 ml of the scrambled shControl or shMELK lentiviruses was incubated with BGC823 and SGC7901 cells overnight at 37°C in a humidified cell culture incubator. Stable GC cells with depleted endogenous MELK expression were selected by culturing in puromycin (0.8 μg/ml).

### Cell proliferation assay

Cell proliferation was measured using a Cell Counting Kit-8 (CCK-8) (Dojindo, Japan) according to the manufacturer's protocol. 2×10^3^ cells/well for BGC823 and SGC7901 were incubated in 96-well plates (Corning-Costar, NY, USA) for 24, 48, 72, and 96 hours. 10 μl of the CCK-8 solution was added to each well and the plates were incubated for 2 more hours at 37°C. Absorbance values of all wells were then measured with a reference wavelength of 450 nm in a Microplate Reader (Bio-Rad, USA).

### Cell migration and invasion assay

Cell migration was assessed with a wound-healing assay. BGC823 and SGC7901 cells were incubated in a 6-well plate (Corning-Costar, NY, USA), and the confluent cell surface was then scratched with a pipette tip. The wound width was recorded using a microscope at 24 and 48 hours. For trans-well chamber-based migration and invasion assays, 5×10^4^ cells were loaded into an insert, provided with serum-free medium, and allowed to pass through a polycarbonate filter, which had been either pre-coated with 100 μg of Matrigel (Becton Dickinson, San Jose, CA) for the invasion assay or left uncoated for the migration assay. The lower chambers were filled with DMEM and 10% FBS. Cells on the upper surface of the filters were wiped out after 24 hours (migration assay) or 72 hours (invasion assay). The membranes were fixed with methanol for 10 minutes and stained with 0.5% crystal violet for 10 minutes. The cells on the underside of the filter were counted in five randomly selected microscopic views.

### Western blot analysis

The protein expression levels of GC cell lines and primary GC tissues were analyzed by Western Blotting. Cells were lysed in pre-chilled RIPA lysis buffer (Pierce Biotechnology, Rockford, IL) containing protease inhibitor cocktail (Roche, Basel, Switzerland) for 30 minutes, then centrifuged at 15,000 g for 20 minutes. 50 μg of protein extract were separated by 10% SDS polyacrylamide gel electrophoresis, and transferred onto a 0.45 μm polyvinylidene difluoride (PVDF) membrane (Whatman, Germany). The membrane was blocked for 1 hour at room temperature with blocking buffer (pH 7.6) containing 5% nonfat dry milk, then membranes were incubated with primary antibodies diluted in blocking buffer at 4°C overnight. The antibodies against MELK (Sigma-Aldrich, St. Louis, MO, USA), E-cadherin, N-cadherin, Akt, and Snail were all diluted at 1:1000.

### Flow cytometric analysis

For cell cycle analysis, cells near 50% confluence were synchronized in the G_0_/G_1_ phase by overnight incubation in serum-free medium. Cells were then incubated in the complete medium containing 25 nM or 50 nM OTSSP167. After 24 hours (BGC823) or 18 hours (SGC7901) of incubation, the cells were trypsinized, washed with PBS, and fixed with 70% ethanol for 16 hours at −20°C. The samples were washed with PBS and stained with PI/RNase Staining Buffer (BD Biosciences) for 15 minutes. Cell cycle analysis was performed by fluorescence flow cytometry on a FACScan machine (BD Biosciences). For apoptotic analysis, cells were stained using an Annexin V/PI double staining kit (DOJINDO, Japan) according to the manufacturer's protocol.

### *In vivo* mouse models of gastric cancer cell lines

Animal studies were carried out in strict adherence with institutional guidelines. BGC823-shMELK cells and BGC823-shControl cells (approximately 1.5 × 10^6^ cells in a 200 μl volume per mouse) were injected into the right hind legs of 6-week-old BALB/c-nude mice (10 mice total, 5 mice per group). Tumor growth was monitored three times a week by measuring the width and length of the tumors with calipers. The tumor volume was calculated by the formula V= 0.5 ×L ×W^2^.

BGC823 cells with or without MELK silencing (0.5 × 10^6^ cells in a 100 μl volume per mouse) were injected intravenously via a 30-gauge needle inserted into the tail vein of female BALB/C-nude mice (5-6 weeks old). Six weeks later, the mice were sacrificed, and the lungs were removed and fixed with Bouin's fixative. The presence of lung metastases was evaluated at autopsy.

### Establishment of GC-patient derived tumor xenograft mouse model bank

Surgically removed GC tissues were obtained from Peking University Beijing Cancer Hospital with the approval of Institutional Review Boards, and an informed consent document was signed by patients, which covered the use of tumor material for research purposes. Tumors were delivered directly from the operating room to the laboratory in culture media (DMEM with 500 units/ml penicillin and 500 ug/ml streptomycin). Pieces of non-necrotic tissue were chosen and minced into 1-3 mm^3^ pieces with ophthalmic scissors under sterile conditions. Minced tumor pieces and Matrigel (BD Biosciences) were mixed at a ratio of 1:1, then 100 ul of tumor tissue homogenate was injected into the groin of each F1 generation mouse (4- to 6-week old female NOD/SCID (Beijing HFK Bioscience Co., Beijing, China)) subcutaneously using an 18 gauge needle. All procedures were completed within 1 hour after surgical specimens were harvested and placed on ice. The mice were maintained under pathogen-free conditions and a 12 hour light/dark cycle. When the tumors reached an approximate size of 1000 mm^3^ (1/2 length × width^2^), they were harvested, minced, and reinjected into F2 generation mice using the same method described for F1 mice.

A section of the tumor from each generation was fixed in 10% formalin and subsequently embedded in a paraffin block. All the tumor specimens, including the patient's primary tumor, were stained with hematoxylineosin (H & E) and examined microscopically. Comparisons between primary tumor and TumorGraft samples were made by pathologists in order to exclude spontaneous lymphomas in the NOD/SCID mice.

### Evaluation of antitumor activity

MELK-positive and -negative GC-PDX mouse models were randomly selected according to the results of IHC performed on the original GC and TumorGraft tissues. When tumor volume reached 100-200 mm^3^, the mice were randomly assigned to treatment and control groups and dosing was initiated. OTSSP167 was administered at 15 mg/kg intravenously to the third-generation mice once every two days for 2 weeks. The control group was treated with vehicle (PBS) in the same way. Tumor size was monitored every two days by caliper measurements. The weight of the mice was also measured as an indicator of treatment toleration. Tumor growth inhibition (TGI) was assessed in accordance with the formula {1–(T–T0) / (C–C0)} × 100, where T and T_0_ are the mean tumor volumes at the end of the drug administration and day 0, respectively, for the treated group, and C−C_0_ are those for the vehicle control group.

### Statistical analysis

Chi-square tests were used to compare the differences in MELK protein expression. The overall survival (OS) curve was calculated with the Kaplan-Meier method and analyzed with the log-rank test. Relative risks (RRs) of death associated with MELK expression and other predictor variables were estimated by the univariate Cox proportional hazards model. Multivariate Cox models were also constructed to estimate the RR for MELK expression. All statistical analyses were carried out using SPSS statistical software (version 18.0; SPSS Inc., Chicago, IL, USA). A two-tailed *p*-value less than 0.05 was considered statistically significant.

## SUPPLEMENTARY FIGURES AND TABLE


