# Supplementary material for: Maternal embryonic leucine zipper kinase serves as a poor prognosis marker and therapeutic target in gastric cancer
Source: Oncotarget. 2015 Dec 19;7(5):6266–80. doi: 10.18632/oncotarget.6673 (PMC4868755; doi:10.18632/oncotarget.6673)
Supplement: Supplementary file 1 [file oncotarget-07-6266-s001.pdf]

SUPPLEMENTARY FIGURES AND TABLE

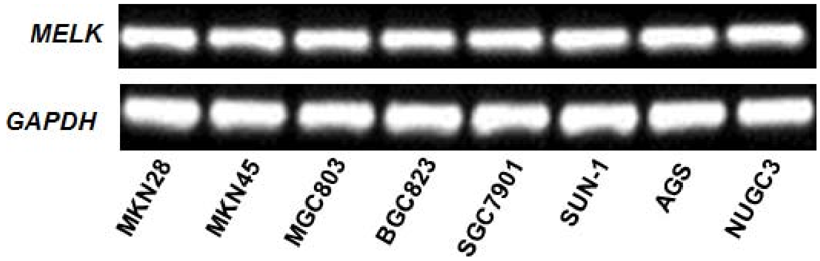

Supplementary Figure S1: *MELK* expression in GC cell lines by RT-PCR.

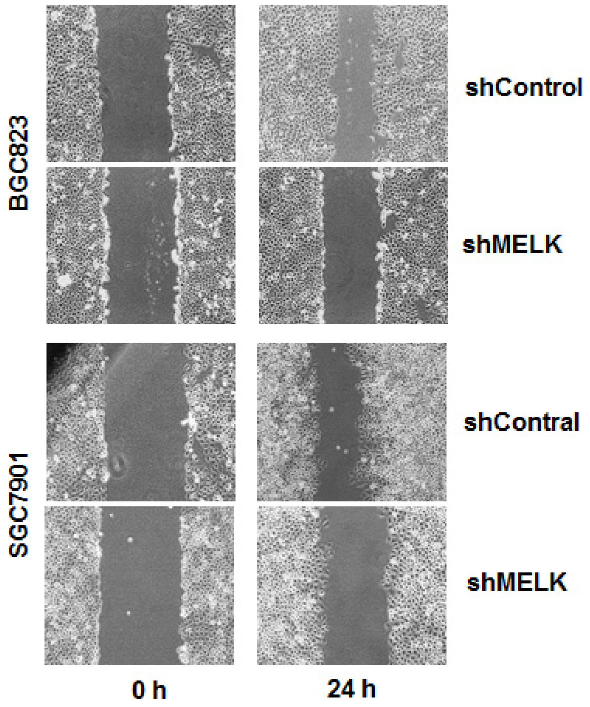

Supplementary Figure S2: Wound-healing assay.

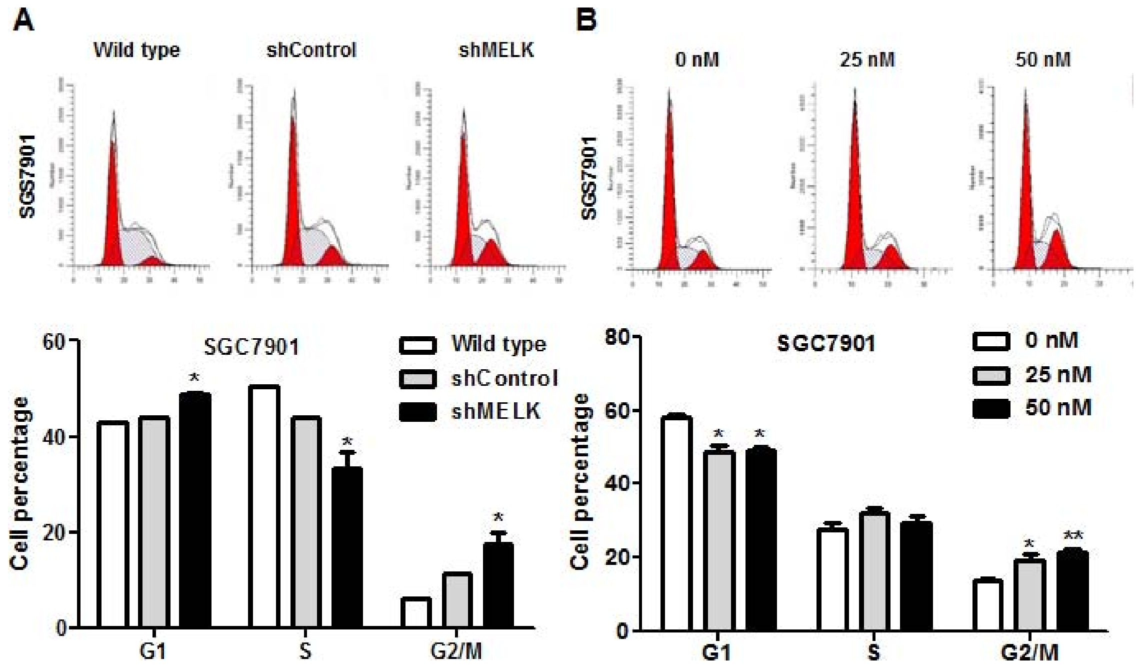

Supplementary Figure S3: Cell cycle analysis by flow cytometry.

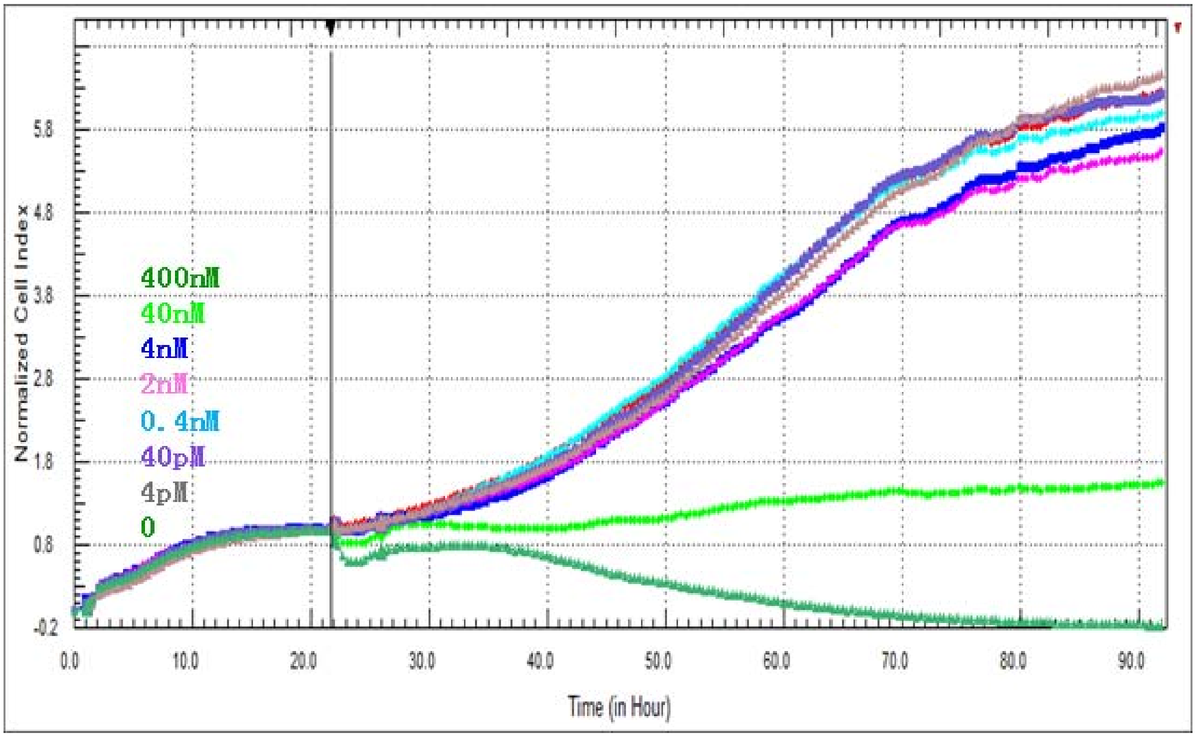

Supplementary Figure S4: Cell proliferation assay by RTCA.

Supplementary Table S1: Expression of MELK in GC and adjacent noncancerous gastric tissues

Variable	MELK expression		P^a value
	Negative, n (%)	Positive, n (%)	
Gastric cancer tissues	33 (31.7)	71 (68.3)	0.036
Adjacent noncancerous tissues	74 (71.2)	30 (28.8)	

[a] Chi-square test
